# Clinical judgment of the need for professional mental health care in patients with cancer: a qualitative study among oncologists and nurses

**DOI:** 10.1007/s11764-021-01151-2

**Published:** 2021-12-02

**Authors:** Jeanet F. Karchoud, Anja J. Th. C. M. de Kruif, Femke Lamers, Myra E. van Linde, Joyce M. van Dodewaard-de Jong, Annemarie M. J. Braamse, Mirjam A. G. Sprangers, Aartjan T. F. Beekman, Henk M. W. Verheul, Joost Dekker

**Affiliations:** 1 Department of Psychiatry, Amsterdam University Medical Centers, Vrije Universiteit, Amsterdam Public Health Research Institute, PO Box 7057, 1007 MB Amsterdam, the Netherlands; 2 Department of Epidemiology and Biostatistics, Amsterdam University Medical Centers, Vrije Universiteit, Amsterdam Public Health Research Institute, Amsterdam, The Netherlands; 3 Department of Medical Oncology, Amsterdam University Medical Centers, Vrije Universiteit, Cancer Center Amsterdam, Amsterdam, the Netherlands; 4 Department of Medical Oncology, Meander Medical Center, Amersfoort, the Netherlands; 5 Department of Medical Psychology, Amsterdam University Medical Centers, University of Amsterdam, Amsterdam Public Health Research Institute, Amsterdam, the Netherlands; 6 Department of Medical Oncology, Radboud University Medical Center, Nijmegen, the Netherlands

**Keywords:** Judgment, Oncologist, Nurse, Mental health care, Cancer

## Abstract

**Purpose:**

In daily practice, oncologists and nurses frequently need to decide whether or not to refer a patient for professional mental health care. We explored the indicators oncologists and nurses use to judge the need for professional mental health care in patients with cancer.

**Methods:**

In a qualitative study, oncologists (*n* = 8) and nurses (*n* = 6) were each asked to select patients who were or were not referred for professional mental health care (total *n* = 75). During a semi-structured interview, they reflected on their decision concerning the possible referral of the patient. Thematic analysis was used to analyze the data.

**Results:**

Respondents reported using a strategy when judging whether professional mental health care was needed. They allowed patients time to adjust, while monitoring patients’ psychological well-being, especially if patients exhibited specific risk factors. Risk and protective factors for emotional problems included personal, social, and disease- and treatment-related factors. Respondents considered referral for professional mental health care when they noted specific indicators of emotional problems. These indicators included lingering or increasing emotions, a disproportionate intensity of emotions, and emotions with a negative impact on a patient’s daily life or treatment.

**Conclusions:**

This study identified the strategy, risk and protective factors, and the indicators of emotional problems used by oncologists and nurses when judging the need for professional mental health care in patients with cancer.

**Implications for Cancer Survivors:**

Oncologists and nurses can play an important role in the identification of patients in need of professional mental health care.

**Supplementary Information:**

The online version contains supplementary material available at 10.1007/s11764-021-01151-2.

## Introduction

Cancer occurrence is a major stressor in patients and results in a wide range of emotions. Most patients are able to manage these emotions with support from relatives, friends, and caregivers [1–4]. However, some patients have difficulty dealing with the emotions triggered by a traumatic event such as the diagnosis of cancer, illustrated by the increased incidence of mental disorders such as mood or anxiety disorders [5–7]. These patients may require professional mental health care to help them cope.

Several screening instruments have been developed to help identify patients likely to require professional mental health care, one example being the Distress Thermometer [8]. However, it has been consistently observed that the majority of patients scoring above the cutoff for distress subsequently decline professional mental health care [9–12]. Typically, while over 30–40% of patients score above the cutoff for distress [12, 13], only around 10–15% of patients report a need for professional mental health care [7, 12, 14]. It has been argued that this discrepancy is either due to suboptimal implementation of distress screening and referral programs or to mental health care-related stigma [15]. While these factors may play a role, the underlying problem seems more fundamental in nature. Many patients report that they do not need professional mental health care but instead prefer support from family, friends, and/or clinicians to help them deal with the emotions associated with cancer and its treatment [12]. In addition, from a theoretical perspective, a distinction should be made between adaptive and maladaptive emotions [12]. Emotions are essentially adaptive—they help us adjust to events in the environment, such as the diagnosis and treatment of cancer [16]. Equally, emotions can sometimes hamper adaptation, leading to significant distress and disability. Emotions are maladaptive if they are disproportionally severe or persistent, and if they interfere with functioning [17]. Professional mental health care is indicated only in the event of maladaptive emotions [12]. In light of these considerations, we can question the value of distress screening instruments. While these instruments are appropriate for determining distress, they are not designed for determining whether patients’ emotions are maladaptive, and they appear less well-suited to determining the need for professional mental health care. This conclusion highlights a need for additional indicators that can reliably distinguish those patients who genuinely require professional mental health care.

In daily practice, oncologists and nurses play a key role in identifying patients who need professional mental health care and frequently need to decide whether or not to refer a patient. We therefore expected that this clinical experience may have led to valuable, intuitive knowledge concerning indicators of emotional problems that require professional mental health care. To detect distress, oncologists and nurses reportedly look for affective, verbal, and physical indicators of distress, using techniques such as getting to know the patient, intuition and subjective judgment, as well as familiarity with the patient’s medical history [18, 19]. However, the specific indicators that are used to judge the need for professional mental health care are unknown. The qualitative study described here aimed to explore which indicators oncologists and nurses use when judging the need for professional mental health care in patients with cancer.

## Methods

### Setting, design, and research team

This explorative study was conducted at departments of medical oncology in two academic hospitals and one teaching hospital in the Netherlands between September 2019 and January 2020. Applying a qualitative study design [20], a researcher with experience in qualitative research methods (JK) interviewed oncologists and nurses. She was supervised by a researcher specialized in qualitative research methods (AdK) and other members of the team with backgrounds in psychology, psychiatry, medical oncology, and epidemiology. Throughout the study, the researcher made notes in a logbook, which were critically discussed with the supervisors.

### Procedure and data collection

The researcher initially presented the study plan at a staff meeting (of oncologists or nurses) in the departments participating in the study. Oncologists and nurses who expressed interest were provided with further information and received a written invitation to participate. All participants confirmed their consent in writing. The aim was to recruit six medical oncologists and six nurses. Participants were told that they would be interviewed regarding their considerations when deciding if a patient should be referred for professional mental health care. To thoroughly explore these considerations, they were asked to prepare for the interview by selecting three of their cancer patients who were referred for professional mental health care and three who were not referred. Purposive sampling based on age and gender was encouraged to maximize the diversity of patients, as these factors are known to be associated with emotional responses to cancer.

The researcher did not know the clinicians she interviewed. The interview was conducted at the clinician’s office, and during the interview, respondents reflected on their decisions concerning referral or non-referral of their patients for professional mental health care. Interviewees had access to electronic patient files which included notes on patients’ psychosocial well-being and care needs. While reviewing these notes, the researcher prompted respondents to “think aloud” concerning why they felt that the patient needed or did not need professional mental health care [21]. The interviewer used a topic list to guide the interview [18, 19, 22–24] (see Supplementary file 1). One interview was interrupted and was repeated at a later moment.

We expected that six medical oncologists and six nurses, each reporting on six patients, would result in useful clinical information, while with the expected total of 72 interviews (= 12 clinicians *6 interviews each), we would not exceed our capacity to analyze interviews.

All interviews were audio-recorded, and field notes were taken after the interview. The duration of the interviews was approximately 1 h on average. Data collection ended when data saturation was achieved (at the patient level). The research team’s conclusion that data saturation had been achieved was based on a systematic discussion of the data, including newly collected data.

### Data analysis

Data collection and analysis constituted an iterative process. The interviews were transcribed verbatim by a research assistant, and the interviewer ensured the accuracy of the transcription. The researchers analyzed the transcribed interviews using thematic analysis [25]. The six steps in the analysis comprised (1) becoming familiar with the data; (ii) generating initial codes; (iii) searching for themes; (iv) reviewing themes; (v) defining and naming themes; and (vi) producing the report [25–27]. The qualitative data analysis software MAXQDA was used.

**Fig. 1:**
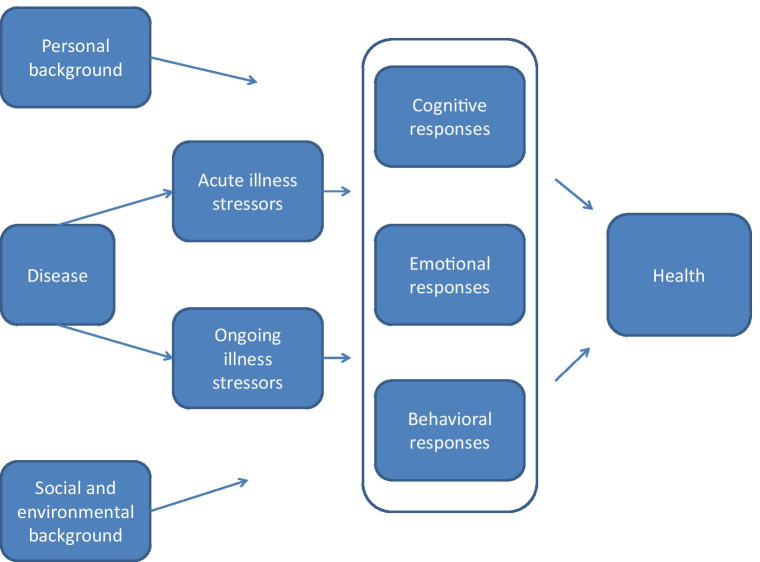
Model of psychological adjustment to chronic disease (reproduced from Disability and Rehabilitation [28])

Two researchers (JK, AdK) analyzed the data independently and any discrepancies were discussed until an agreement was reached. The data analysis was repeatedly discussed with the research team, and various conceptualizations of codes and themes were discussed and justified among the research team. During analysis, the need arose for a model to organize the codes, and the model of psychological adjustment to chronic disease was chosen as a sensitizing concept to help organize the codes [28]. This model distinguishes the patient’s personal, social, and environmental background from possible emotional, cognitive, and behavioral responses to disease or stressors (see Fig. 1). Below, the codes that concerned the patient’s personal, social, and environmental background are referred to as “risk and protective factors,” while the codes that concerned emotional, cognitive, and behavioral responses to disease/stressors are referred to as “indicators for a referral” (see Results section). The coding system is described in detail in Supplementary file 2.

## Results

### Participants

Eight oncologists and six nurses participated in the study (*n* = 14). Their mean age was 46.4 (SD 9.1) years; 11 were female, 3 male; 10 worked in an academic setting, including 4 in a teaching hospital; participants had 12.64 (SD 6.36) years of clinical experience in oncology; 7 had taken a course or had training in patients’ psychosocial well-being while 7 had not. None of the departments of medical oncology involved in the study had implemented a procedure for systematic distress screening using the Distress Thermometer/Problem List or another instrument. Oncologists and nurses relied on their clinical judgment to identify patients in need of referral for professional mental health care. Two oncologists and two nurses reported that they had occasionally used the Distress Thermometer/Problem List.

Participants selected a total of 75 patients. The mean age of patients was 58.4 (SD 11.4) years; 37 were female, 38 male; their diagnoses were colorectal (*n* = 31), skin (*n* = 5; melanoma), ovarian (*n* = 2), pancreas (*n* = 5), bladder (*n* = 2), head and neck (*n* = 7), stomach (*n* = 2), testicle (*n* = 3), breast (*n* = 8), and prostate (*n* = 2) cancer (no information on diagnosis: *n* = 8). Patients were treated with curative intent (*n* = 19) or in a palliative setting (*n* = 40) (no information: *n* = 16). Clinicians selected more patients who were referred for professional mental health care (*n* = 50) than patients who were not referred (*n* = 25), as they considered referred patients to be more informative concerning indicators for referral than non-referred patients.

### Strategy, risk and protective factors, and indicators

**Fig. 2:**
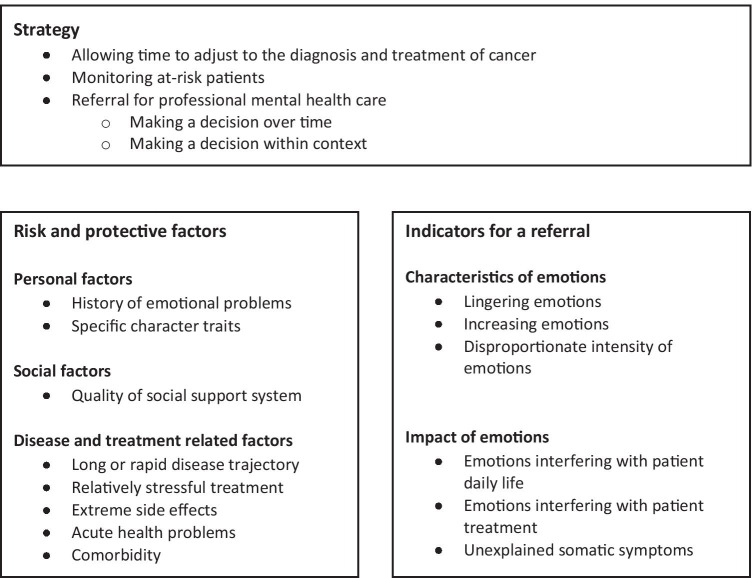
Clinical judgment of the need for professional mental health care in patients with cancer

The analysis revealed three main themes in the oncologists’ and nurses’ reflections: (1) a strategy to judge whether professional mental health care was needed, (2) risk and protective factors for the development of emotional problems, and (3) indicators for referral. Figure 2 provides an overview and Table 1 provides examples of respondents’ comments. Overall, oncologists and nurses mentioned comparable strategies, factors, and indicators, although specific examples varied. One oncologist and two nurses mentioned that nurses usually have more time than oncologists to evaluate a patient’s personal and social background.

**Table 1:** Themes and examples of quotations

Theme	Example of quotation
Strategy	
1. Allowing time to adjust to the diagnosis and treatment of cancer	“I don’t immediately refer patients for professional mental health care. It is a process. I first watch how they cope with their disease over time.” (Respondent 1, oncologist) “When people have just received a diagnosis, needing help is often not yet an issue. At that point it is just a matter of beginning and seeing how things go. However, right at the first meeting I always say: 'know that if you can't figure it out or if you feel like you need help, we can provide it.” (Respondent 14, oncologist) “You try to be as accessible as possible to people; you do your best to encourage them: 'You can always ask anything, you can say anything, nothing is crazy. You will only be doing yourself an injustice if you don't.' By creating this sense of security, you hope that people also will sense the invitation.” (Respondent 4, nurse)
2. Monitoring at-risk patients	“At the beginning it may already be evident which patients are at risk and may need professional mental health care. When we notice this, we start by providing extra support, such as calling between appointments.” (Respondent 11, nurse) “This very young woman received bad news again and again, and also had young children. Yes, in such cases patients themselves often ask for guidance.” (Respondent 2, nurse)
3. Referral for professional mental health care: making a decision over timing and within context	“You see so many patients in stressful situations, at a certain point you get a frame of reference as to what is ‘normal’ when responding to bad news.” (Respondent 14, oncologist)
Risk and protective factors	
***Personal factors***	
1. History of emotional problems	“I always check someone’s mental health history. When patients have already had a previous burnout or depression, these are triggers for me that they might need professional mental health care again. You keep an eye on them.” (Respondent 11, nurse)
2. Specific character traits	“When someone is generally anxious, without being pathologically anxious in daily life, and ends up in such a difficult situation, these feelings could develop into excessive fear and stress. In that case they may benefit from professional mental health care.” (Respondent 8, oncologist)
***Social factors***	
1. Quality of social support system	“It makes sense that patients with cancer become emotional. The best scenario is when the people around them provide support. If you see that this is not working, they may need more support through professional mental health care.” (Respondent 8, oncologist)
***Disease- and treatment-related factors***	
1. Long or rapid disease trajectory 2. Relatively stressful treatment 3. Extreme side effects 4. Acute health problems 5. Comorbidity	“When there is rapid progression everything is going so fast that it is difficult for patients to make the necessary mental adjustments. Reality catches up with them and they might need professional mental health care to help them process it.” (Respondent 2, nurse) “Psychological treatment was requested because I was just struck by the whole course: a year long, with very serious, actually really serious, symptoms” (Respondent 6, oncologist) “The reason for referral is that the patient also has < …. > disease, and has been in a diagnostic trajectory for that for a very long time" (Respondent 4, nurse)
Indicators for referral	
***Characteristics of emotions***	
1. Lingering emotions	“Some people are extremely sad when they get their diagnosis. That’s normal, they need time to process. It becomes problematic when patients experience lingering sadness. That is a reason to refer patients for professional mental health care.” (Respondent 10, oncologist)
2. Increasing emotions	“But then again, she was emotional, well, you just noticed over time that it didn't get better but actually got worse. And then she also became more emotional during visits, whereas she always was a little bit already, but it did increase.” (Respondent 3, oncologist)
3. Disproportionate intensity of emotions	“For me a trigger is when I see very little emotion after patients receive bad news. The patient seems undaunted while I have just exploded a nuclear bomb: it doesn’t add up.” (Respondent 14, oncologist)
***Impact of emotions***	
1. Emotions interfering with patient’s daily life	“Patients need professional mental health care when they are no longer engaging in any activities due to anxiety or depressive feelings. Of course, there are limitations to what is possible, although a lot is still possible to various extents. For example, taking a walk rather than just sitting on the couch at home.” (Respondent 1, oncologist)
2. Emotions interfering with patient treatment	“There are always certain patients who become known to the whole department, even when he or she is not their own patient. This is often a sign that professional mental health care is needed.” (Respondent 1, oncologist)
3. Unexplained somatic symptoms	“Explaining the origin of a physical complaint is always important, as there could be underlying emotional reasons that require professional mental health care.” (Respondent 5, nurse)

### Strategy

Respondents gave patients the opportunity to adjust to the diagnosis and treatment of cancer. Rather than immediately offering professional help, patients were first allowed time to deal with their emotions and were monitored regarding psychological well-being. Monitoring consisted of note-taking in the patient record, together with observation and interviews with the patient. Risk and protective factors were taken into account by respondents when judging whether professional mental health care would be needed. Respondents stated that they became more attentive when they noticed risk factors because patients exhibiting risk factors were considered more likely to experience emotional problems, compared to patients without risk factors. Some respondents informed at-risk patients that professional mental health care would be available if needed. Other respondents mentioned providing additional support to at-risk patients, for example through more frequent calls or visits.

A decision to refer a patient for professional mental health care developed gradually rather than at one specific moment, and this decision was made within the context of risk and protective factors. When judging timing and context, respondents relied on their experience with other patients, who served as a reference standard and on personal considerations.

### Risk and protective factors

Respondents mentioned several factors associated with risk for emotional problems that require professional mental health care, or conversely, protect patients from problems. The absence of a protective factor was frequently seen as a risk factor, and vice versa. We categorized these as personal, social, and disease- and treatment-related factors.

#### *Personal factors*

Personal risk factors included a history of emotional problems, such as earlier treatment by a psychologist, a previous episode of burnout, depression, or emotional problems due to an interpersonal conflict. Respondents suspected that such factors predisposed patients to cancer-related emotional problems.

Respondents mentioned specific character traits as risk or protective factors for emotional problems. They reported that professional mental health care is more often needed when patients are generally anxious or hyperactive, tend to worry, or tend to need to keep everything under control. Respondents expected patients to be better able to handle their emotions when they were generally calm, optimistic, or realistic. Some respondents also mentioned that a positive sign is the overall motivation of a patient, for example toward their treatment or toward maintaining a healthy lifestyle.

#### *Social factors*

Respondents highlighted the importance of patients’ social support system and regarded a good social support system as a protective factor. When patients are surrounded by friends or family with whom they have good, stable relationships and who provide support, respondents felt that professional mental health care was less likely to be needed. Conversely, respondents mentioned a weak social support system as a risk factor and suspected that patients would have difficulty handling their emotions without close friends or family, if there was tension or conflict with friends or family, or when others in their social circle were also having a difficult time.

#### Disease- and treatment-related factors

Disease or treatment that was comparatively stressful was considered by respondents a risk factor for emotional problems. These included long or rapid disease trajectories, a relatively stressful treatment, extreme side effects, acute health problems, or comorbidity. Respondents believed that these types of adverse situations are difficult to adapt to and could eventually lead to a need for professional mental health care.

### Indicators for referral

Respondents reported that specific characteristics of emotions and a negative impact of emotions were indicators for referral to professional mental health care.

#### Characteristics of emotions

Patients were referred to professional mental health care when respondents noticed specific emotional characteristics. These included lingering emotions such as persisting anxiety, anger, sadness, or stress. Some respondents also referred patients if they gradually became more emotional over time. A decreasing intensity of emotions was regarded as a sign of good mental health.

Referral of patients was often prompted when the intensity of emotions was considered disproportionate to the patient’s situation. Descriptions of disproportionate emotions included intense depressive feelings, excessive crying or sadness, extreme panic, severe anxiety, or experiencing many different emotions concurrently. Some respondents emphasized that intense emotions were to be expected in a stressful situation and that these did not immediately require a referral for professional mental health care. Emotions were also considered disproportionate when patients showed little or no emotion in a stressful situation.

#### Negative impact of emotions

Respondents mentioned weighing the impact of emotions on a patient’s daily life and activities. Examples of indicators for referral included sleep problems due to anxiety and emotions that prohibit patients from reintegrating into daily life or engaging in activities. Respondents saw reintegration and activities as positive indicators of a patient’s mental health, regardless of the specific activity (work or leisure activities).

Patients were referred to professional mental health care when their emotions appeared to interfere with medical treatment by influencing aspects such as communication and decision-making. Professional mental health care was also considered indicated when patients were felt to be “claiming” medical personnel through behavior such as repeated phone calls to confirm information or decisions.

Finally, respondents mentioned unexplained somatic symptoms as a reason for referral. When physical symptoms could not be explained on medical grounds, respondents were aware that there might be an underlying emotional cause.

## Discussion

Oncologists and nurses described a strategy to judge their patients’ professional mental health care needs. This strategy consisted of allowing patients time to adjust to stressful events while monitoring their psychological wellbeing, especially when patients exhibited specific risk factors. If emotional problems were noticed, patients were referred to professional mental health care. This strategy was not explicit; instead, it was an implicit strategy that emerged from the interview data. This strategy can be characterized as “watchful waiting,” i.e., closely monitoring a patient’s condition and only providing treatment when justified by symptoms [13, 29, 30].

Emotions, as such, were not considered a symptom requiring professional mental health care. When patients experienced intense emotions, oncologists and nurses did not immediately make a referral, only referring patients if emotions were considered to constitute a problem. This approach implies that they made a distinction between adaptive and maladaptive emotions [12]. In the field of mental health, emotions are considered essentially adaptive—they help people to adapt to events in their environment, such as the diagnosis and treatment of cancer [16]. Sometimes, emotions become maladaptive, hampering adaptation and leading to significant distress and disability. Emotions are considered maladaptive if they are disproportionally severe or persistent, and if they interfere with functioning [17]. Although oncologists and nurses did not articulate this distinction as such, their clinical behavior implies that they intuitively distinguished between adaptive and maladaptive emotions; that is, distinguishing emotions that don’t require professional mental health care from those that do. Elsewhere, we reported further evidence that respondents make this distinction: oncologists’ and nurses’ notes in the patient file better corresponded to a patient’s experienced need for mental health care than to experienced distress. This suggests that clinicians took notes if they thought emotions were maladaptive and required mental health care, whereas few notes were taken in the case of adaptive emotions that did not justify treatment [31].

Mental health research concerning indicators that distinguish adaptive from maladaptive emotions is in a rather early stage. These indicators can be found in various research areas, including emotional dynamics [32], emotion regulation [33], life goals, and subjective well-being [34], as well as the network theory on mental disorders [35]. In the field of psycho-oncology, distinguishing between adaptive and maladaptive emotions is a rather novel concept [12, 36]. The dominant opinion at present is that intense emotions need to be treated [37]. In contrast, our study shows that oncologists and nurses consider emotions to be a normative aspect of the diagnosis and treatment of cancer (see also [18]). Emotions were considered problematic only if emotions lingered, increased over time, or were disproportionately intense, with a negative impact on a patient’s daily life or treatment. This novel and helpful information can be used to develop operational indicators to help distinguish those patients who need professional mental health care.

The risk and protective factors for emotional problems found in this study are generally in line with empirical literature. All factors reported by our respondents are supported by empirical evidence – a history of emotional problems, specific character traits, the quality of the social support system, and disease- and treatment-related factors [38–42]. Apparently, clinical experience and/or formal training have led to a good understanding of risk and protective factors among our respondents. However, the literature also includes evidence for other factors that were not mentioned by our respondents, such as sleep disturbance as a risk factor for emotional disorders [38].

A number of methodological issues need to be considered when interpreting the results of this study. First, two levels of data can be distinguished in the current study: the clinician level (oncologists and nurses) and the patient level. The fourteen contributing clinicians varied in gender, age, clinical experience, and academic or teaching hospital setting, as well as in participation in courses or training concerning the psychosocial well-being of patients. Although this group of oncologists and nurses was diverse, it cannot be excluded that other clinicians would have mentioned additional themes (in other words, at the clinician level we can’t confirm that data saturation was achieved). Further research among oncologists and nurses is therefore indicated. At the patient level, 75 patients were purposefully selected to vary in cancer diagnosis, gender, and age. Data saturation was achieved at this level. Second, we used the “think aloud” method to describe how clinicians judge the need for professional mental health care in patients with cancer. This study did not allow conclusions about how consistently this approach was applied. It is quite conceivable that in a busy practice, it is not always possible to consistently assess all patients. Moreover, clinicians likely differ in their approach, and while some may be interested and experienced in judging the need for professional mental health care, others may be less interested or experienced. Taking a broader view, another factor worth considering is that clinicians in different countries may vary in their approach, reflecting differences in clinical practice from country to country. Further research in more countries is needed in order to determine to which extent clinicians make use of the strategy, the risk and protective factors, and the indicators of emotional problems described here. Both intra- and inter-clinician differences should be evaluated. Third, a range of measures was taken in order to ensure the trustworthiness of data [27]. Regarding reflexivity: the researcher documented theoretical and methodological decisions and their rationale, as well as decisions regarding the analysis. Decisions were based on a critical discussion with the supervising team. With regard to credibility: we applied researcher triangulation, and the oncologists and nurses applied purposive sampling to select a variety of patients based on age and gender. However, this may have introduced bias, as oncologists and nurses relied on memory to select patients. A summary of the interviews was not returned to participants to check for accuracy (member check), due to clinicians’ busy schedules. With regard to transferability: our sample was diverse, at both the clinician and patient levels, and we described the relevant characteristics of departments, clinicians, and patients (thick description). With regard to dependability and audit trails: data collection and data analysis occurred in an iterative manner, and we provided a detailed account of the various steps of the study.

Current recommendations regarding the management of emotions in patients with cancer involve distress screening and providing a referral for psychosocial care if needed [37]. Unfortunately, screening and referral programs have shown a limited effect on the psychological well-being of patients [13, 43]. As an alternative to screening and referral, it has been suggested that oncologists and nurses can play an important role in the management of patients’ emotions and the identification of patients in need of professional mental health care [12, 15]. The results of the present study provide preliminary support for this idea. The reported strategy of watchful waiting, the risk and protective factors, and the indicators of emotional problems that require professional mental health care are commensurate and appropriate. However, further development and evaluation of this approach is certainly required. Assuming that not all clinicians are currently able to provide adequate clinical judgment, additional training and organizational measures may help these clinicians improve. Research findings and patient perspectives in the field of mental health could also be integrated into training, to help further improve clinical judgment. Case findings by clinicians could be further improved by repeated assessment of psychosocial needs, and assessment of psychosocial needs could itself be embedded within broader symptom assessments [15].

In conclusion, this study identified the strategy, risk and protective factors, together with the indicators of emotional problems used by oncologists and nurses when judging the need for professional mental health care in patients with cancer. The findings suggest that oncologists and nurses can play an important role in the identification of patients in need of professional mental health care.

## Supplementary Information

Below is the link to the electronic supplementary material.

Supplementary file1 (DOCX 39 KB)

## Data Availability

The data are not publicly available due to privacy or ethical restrictions.
