# Supplementary material for: Clinical judgment of the need for professional mental health care in patients with cancer: a qualitative study among oncologists and nurses
Source: J Cancer Surviv. 2021 Dec 2;17(3):884–93. doi: 10.1007/s11764-021-01151-2 (PMC10209294; doi:10.1007/s11764-021-01151-2)
Supplement: Supplementary file 1 — Supplementary file1 (DOCX 39 KB) [file 11764_2021_1151_MOESM1_ESM.docx]

Version: 01-11-2021

**Clinical judgement of the need for professional mental health care in patients with advanced cancer: a qualitative study among oncologists and nurses**

**Supplementary files**

**Supplementary File 1**

**Topic list**

When do you think a patient needs or does not need professional mental health care?

*How did you get that idea?*

- Reasons

- Decisive factor(s)

*Did the patient’s emotions or physical complaints contribute to this?*

*Nature*

- Frightened, anxious, panic-stricken, nervous, tense

- Depressed, gloomy, listless, apathetic, bored

- Guilt feeling

- Angry/aggressive, irritated

- Wishing to keep control

- Feeling good, cheerful, energetic

- Satisfied, relaxed, calm

- Concentrated, serious

- Physical symptoms/pain without cause

- Crying

- No emotions

*Characteristics of emotions*

- Extreme

- Unstable, stable

- Phasing out

*Did the patient's way of coping with emotions or the disease contribute to this?*

- Negative, distant

- Stuck in emotions

- Acceptance

- Realistic

- Good disease understanding

**Did the patient's behavior contribute to this?**

*In relation to treatment*

- Interference with treatment

- Interference with clinical relationship

- Wrong focus/perspective

- Contact outside consulting hours

- Disagreement with relatives (goes well vs. does not go well)

*In relation to patient’s (daily) life*

- Interference with activities pursuing life goals

- Interference with social functioning

- Social isolation

- Changed appearance

**Did the patient’s personality contribute to this?**

- Personality

- Introvert vs. extravert

- Quiet person

- Sensible person

- Social network (did anyone else influence the decision to refer)

**Supplementary File 2**

**Final code system**

**1. Differences oncologists and nurses**

1.1 Usually nurses have more time than oncologists

**2. Strategy**

2.1 Allowing time to adjust

2.2 Making a decision over time and within context

**3. Risk and protective factors**

3.1 Personal factors

3.1.1 History of emotional problems

3.1.1.1 Visit to psychologist

3.1.1.2 Burn-out

3.1.2 Specific character traits

3.1.2.1 Worrying

3.1.2.2 Hyperactive

3.1.2.3 Anxious

3.1.2.4 Urge to control

3.1.2.5 Realistic

3.1.2.6 Motivated

3.1.2.7 Calm

3.1.2.8 Sober minded

3.1.2.9 Optimistic

3.2 Social context

3.2.1 Social support

3.2.1.1 Good social support system

3.2.1.2 Weak social support system

3.3. Disease and treatment related factors

3.3.1 Extreme side effects

3.3.2 Comorbidity

3.3.3 Relatively stressful treatment

3.3.4 Long or rapid disease trajectory

**4. Indicators for a referral**

4.1 Characteristics of emotions

4.1.1 Intensity

4.1.1.1 Extreme emotions

4.1.1.1.1 No emotions at all

4.1.1.1.2 Very depressed

4.1.1.1.3 Disproportionally anxious

4.1.1.1.4 Severe panic

4.1.1.1.5 A lot of crying

4.1.1.1.6 Extremely angry

4.1.1.1.8 Great sadness

4.1.1.1.9 Many different emotions together

4.1.2 Course of emotions

4.1.2.1 Stable emotions

4.1.2.2 Not getting stuck in emotions

4.1.2.3 Lingering emotions

4.1.2.3.1 Persistent stress

4.1.2.3.2 Always angry

4.1.2.3.3 Persistent anxiety

4.1.2.3.4 Persistent grief

4.1.2.4 Increasing emotions

4.1.2.4.1 More sad

4.1.2.4.2 Increasing stress

4.1.2.4.3 More anxiety

4.1.2.4.4 More fatigue

4.2 Impact of emotions

4.2.1 Emotions interfering with patient’s daily life

4.2.1.1 Fatigue and sleep problems due to emotions

4.2.1.1 Not engaging in activities, no reintegation

4.2.2 Emotions not interfering with patient’s daily life

4.2.2.1 Engaging in activities

4.2.2.1.1 Work

4.2.2.1.2 Leisure

4.2.3 Emotions interfering with patient treatment

4.2.3.1 Treatment impeded by emotions

4.2.3.2 Cannot make decisions

4.2.3.3 Contact with clinical staff

4.2.3.3.1 Claiming

4.2.3.3.2 Search for confirmation

4.2.3.3.3 A lot of (telephone) contact

4.2.4 Unexplained somatic symptoms
